# Electrical impedance tomography system: an open access circuit design

**DOI:** 10.1186/1475-925X-5-28

**Published:** 2006-05-03

**Authors:** Manuchehr Soleimani

**Affiliations:** 1 William Lee Innovation Centre, School of Materials, The University of Manchester, Manchester M60 1QD, UK

## Abstract

**Background:**

This paper reports a simple 2-D system for electrical impedance tomography EIT, which works efficiently and is low cost. The system has been developed in the Sharif University of Technology Tehran-Iran (for the author's MSc Project).

**Methods:**

The EIT system consists of a PC in which an I/O card is installed with an external current generator, a multiplexer, a power supply and a phantom with an array of electrodes. The measurement system provides 12-bit accuracy and hence, suitable data acquisition software has been prepared accordingly. The synchronous phase detection method has been implemented for voltage measurement. Different methods of image reconstruction have been used with this instrument to generate electrical conductivity images.

**Results:**

The results of simulation and real measurement of the system are presented. The reconstruction programs were written in MATLAB and the data acquisition software in C++. The system has been tested with both static and dynamic mode in a 2-D domain. Better results have been produced in the dynamic mode of operation, due to the cancellation of errors.

**Conclusion:**

In the spirit of open access publication the design details of this simple EIT system are made available here.

## Background

The imaging of electrical properties of different materials has been the main topic of many investigations for a number year [1-3]. In EIT, the contrasts in electrical properties, i.e. the conductivity distribution inside an object, is used to generate a tomographic image [2,4]. EIT has potential applications in both medical and industrial fields [5-7]. The advantage of such a technique over more traditional imaging modalities (PET, CT, MRI and ect.) is such that, it provides a non-invasive (or "non-destructive") method and requires no ionizing radiation. Furthermore, EIT is a relatively low cost and simple functional technique. The most significant drawback of EIT is its poor image resolution, which is often restricted by the number of electrodes used for data acquisition. Data acquisition is typically made by applying an electrical current to the object using a set of electrodes, and measuring the developed voltage between other electrodes [2,8,9].

**Figure 1:**
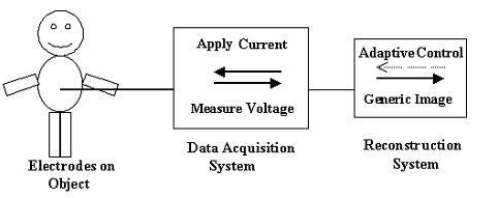
General View of the EIT System.

Figure 1 illustrates a general view of an EIT system. Generally 2-D EIT systems could be categorized into two different sets namely: Applied Potential Tomography (APT) and Adaptive Current Tomography (ACT) [5]. Our instrumentation (SUT-1) is based on single channel measurement technology and so is APT mode.

## Method

The major mathematical modeling of EIT involves calculation of the forward and inverse problems. In the forward problem the governing equations in the EIT field which are derivable from Maxwell's Equations (electrostatic approximation for low frequency) [5] are

     at B (B is the object)     (1)

     P ∈ S (Surface)     (2)

     P ∈ S     (3)

where the *Ũ*(*P*) is the voltage and (*P*) is the specific admittance of B; in which (*P*) = σ(*P*) + *j*ωε(*P*). Equations (1) to (3) are the basic equations used in developing an algorithm to work in an EIT field.

In order to map the resistivity inside a body in a more efficient way, an EIT system SUT-1 has been fabricated [10].

**Figure 2:**
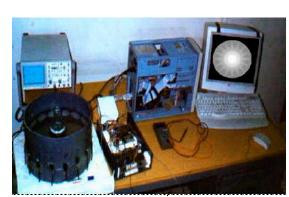
General View of SUT-1 System.

The system has been tested using both 16-electode and 32-electrode modes of operation with different reconstruction algorithms [11]. Figure 2 shows a photograph of SUT-1. Based on this original EIT model, a PC-based system was developed and tested. Note that the system at present is only able to reconstruct images in a 2-D domain.

### SUT-1 hardware

**Figure 3:**
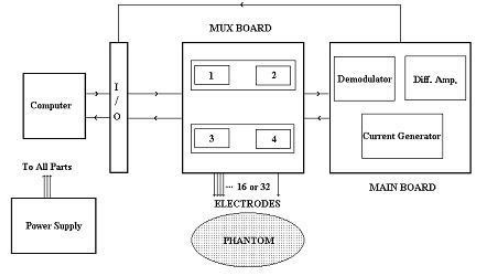
The block-diagram of SUT-1.

**Figure 4:**
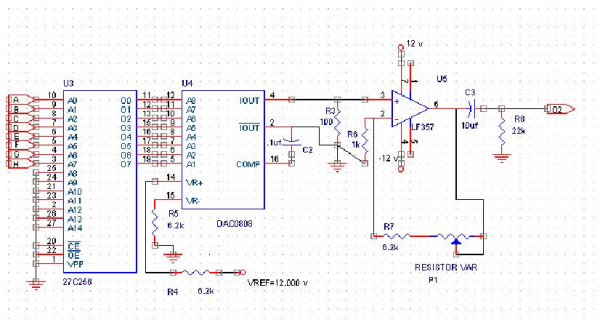
Current Generator.

The block-diagram of SUT-1 is shown in Figure 3. Here only the main blocks of system hardware are discussed. Moreover, for each measuring channel, a well-known block is used [3,12] (figure 4). The utilized computer is a usual Pentium-based PC, which is connected to the measurement system through an Input-Output interface (I/O) card. The main board consists of a current generator with 5 m. A current at 23 kHz and a precision voltage measurement (using synchronized pulse demodulation technique). The accuracy of the digital system is 12 bits. The switching between different pairs of electrodes is carried out by computer using a multiplexer card (MUX board). The collected data from all possible voltage measurements is fed to the image reconstruction software. A brief description of individual modules in this system is given below.

### I/O card

For the the I/O module, an ADVANTECH PCL-812PG I/O card is used [13]. It consists of a 16 bit programmable I/O card with a 12-bit successive approximation analogue to digital converter, (30 kHz sampling rate), programmable Time/Counter/Gain and two 12 bit monolithic multiplying digital to analogue converter output channels. Due to the application of an unsophisticated analogue to digital conversion algorithm, it is not a fast sampling card.

### Current generator

In this module a fixed frequency current source was designed. A detailed diagram of such a current driver is shown in figure 4. For an EIT current generator, the amplitude stability and high output resistance are the most important aspect of the design [14]. Different circuits were built and tested, and finally reached a digital generation method by means of an EPROM (27C258). Furthermore, the EPROM was programmed to produce 256 steps of a 23 kHz sinusoidal waveform. An 8-bit counter was used for reading the EPROM data, and then data were applied to a digital to analogue converter (DAC-0808). The system internal clock ran at 6 MHz. One of the most important advantages of this circuit is related to the synchronous pulses for demodulation, which can be obtained by the address line decoding. Zero crossing point and amplitude peak point can also be determined. The total harmonic distortion (THD) of this current generator is determined to be about 1.3%. The output of this digital oscillator is fed into the current source through a normal gained buffer stage (LF-357). It must be noted that the voltage control current source (VCCS) is a buffered current mirror circuit. We use Analog Devices AD644 are used as the main part and some LF-411 and LF-412 for buffering. The output current is not more than 5 mA.

### Voltage measurement

**Figure 5:**
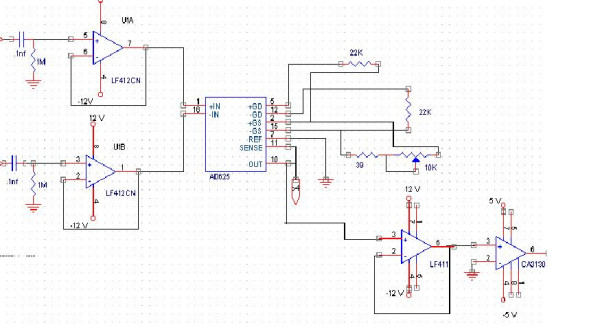
Voltage Measuring Demodulator.

Another important part of the system hardware is the voltmeter. In SUT-1 a synchronous differential demodulator is used. This method is a common method for demodulation in EIT. The noise cancellation capability is one of the important features of this circuit. The circuit diagram of this demodulator is illustrated in figure 5. It is a "Sample and Hold" type of demodulator [15]. An AD-625 instrumentation amplifier is used as the "heart" of the measurement system [16]. The output signal of the demodulator is fed into an I/O card by a controlled gain buffer amplifier block, which uses CA-3130 at the final stage. In the APT mode of operation, the offset and gain error of this stage is of less importance, so, for a better Signal to Noise Ratio, the gain could be increased to a maximum reasonable value. The time duration in which the voltage measurement is performed, is an essential parameter in the overall system speed. This is also dependent upon the multiplexing switching time between different channels. In order to decrease the data reading time, it is possible to use, fast analogue to digital converters, a separate demodulator for each channel and/or a decrease in multiplexing time by means of faster digital switches. For example, in APT mode, 16 electrodes can be directly connected to the 16-bit I/O card for voltage measuring and in this way multiplexing time can be saved and hence errors are reduced.

**Figure 6:**
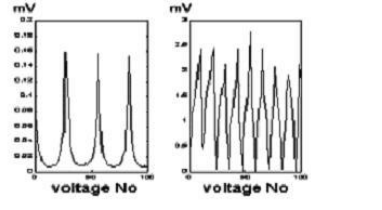
Variation of voltage in measurement module a: adjacent & b: opposite methods for current injection.

We have surveyed different methods namely cross and opposite for data acquisition and their effects on the distinguishability of objects. An example of such methods is shown in Figure 6 in which the variation of measured voltage verses measurement number for a current injector is illustrated.

### Multiplexer

In order to perform data acquisition in 16-electrodes and 32-electrodes mode, a multiplexer circuit is necessary for switching the current injector and voltmeter among the different data channels. Our multiplexer circuit (MUX) consists of four 32 × 1 analogue multiplexers. Each multiplexer is a combination of two 16 × 1 IC-4067 multiplexers. The most significant type of errors arising out of the MUX board, labelled as r_on_, are related to the semiconductor switches, and also cross-talk between different channels. It has to be noted that the r_on _does not have a constant value, but different values for different channels. It is a function of different parameters such as temperature, current, etc in each channel. It is desirable to have the value of r_on _as low as possible.

### Electrodes and phantom

Different cylindrical phantoms are used in this device. In order to simulate behaviour of the human tissue, saline solutions with different concentrations are used. Normal ECG electrodes i.e. Ag-AgCl type, served as an electrical contacting media. Cu electrodes could be another choice for a better and more realistic simulation of electrode-skin contact impedance [17,18].

### Software

**Figure 7:**
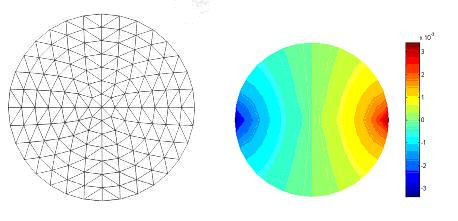
Electric potential distribution with opposite current pattern And 2-D Mesh for FEM Model Used for image.

Here a brief overview of the system software is presented. According to the predefined tasks set by the system hardware design, different programs were developed. In the area of data acquisition, the developed software (system control software) is written using C++ environment. In fact it can be said that this software controls the whole process. However in the field of image reconstruction software is developed using a MATLAB environment. Moreover, a simple program is developed which is capable of generating different meshes for Finite Element Method (FEM). In this F.E. model, triangular elements are used for image reconstruction (see Figure 7). Then, for the image reconstruction in the 32-electrode mode of operation, a modified Newton-Raphson method is implemented.

During APT mode of operation, image reconstruction is performed with 16 electrodes using a Back Projection algorithm and iso-potential lines. Basically this was the "Sheffield Algorithm" with some changes and modifications corresponding to the SUT-1 specification.

## Results

The system performance was tested with different approaches i.e., simulations and real measurement. Some examples of these results are discussed below.

### Results from the simulations

**Figure 8:**
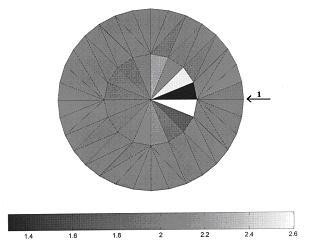
Simulated images due to Electrode positioning error.

**Figure 9:**
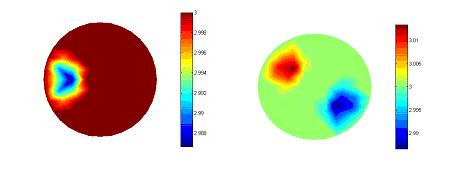
Simulated test reconstructions using regularized Newton-Raphson method.

As described earlier, EIT is very sensitive to different errors. The reconstruction software was tested under different normal conditions and satisfactory results were obtained trough simulations. But abnormal conditions are more important with regard to the overall system performance. One of interesting issue was to observe what would happen if the positions of electrodes were changed. To do this a homogeneous medium is considered. Just one element with different resistivity was implemented at the right side of the second sector. Then a 1 mm error in the exact position of electrode No.1 was introduced. The simulated data was applied to the reconstruction algorithm. The obtained result of the constructed image under this condition is shown in Figure 9. A large error is introduced especially in the adjacent elements as shown on Figure 8. The basic element is seen as black (it has to be white) and this error was propagated through the image. This simulation shows that the mis-positioning of electrodes is one of the important in causing artefacts in medical EIT. Figure 9 shows the reconstruction results from the regularized Newton-Raphson method using simulation data for one and two inclusions.

### Results from the real measurements

**Figure 10:**
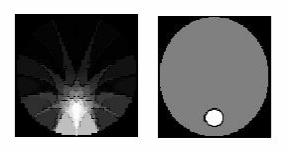
A real test object and its EIT reconstructed image using back projection method.

**Figure 11:**
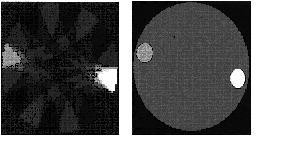
A real test object and its EIT reconstructed image using back projection method.

For this part, the voltage and then reconstructed images are measured in 16 and 32 electrodes modes. In practical conditions, EIT is very sensitive to noise. Electrodes are connected via a shielded cable to the system for noise reduction. Figures 10, 11 illustrate two actual images using a simple phantom in the APT mode. The phantom was made of a PVC cylinder with a 30 cm diameter and filled with saline. Figure 10 shows the design and experimental results for a phantom where an object with different resistivity (a normal milk bottle) is put at the corner, i.e., at x = 0 cm and y = -6 cm from the geometrical centre of the tank.

Figure 10 shows the design and experimental results for a phantom with two objects. Measured data were transferred to the computer, the reconstruction algorithm applied and an image was obtained using the back projection method. As seen in Figure 11, a star artifact resulted from the back projection a well-known artifact for this method without using filters. Basically, it is due to limitation in the number of projections. If the projections number (ray-sum) is increased, the size of this artifact will decrease.

## Conclusion

SUT-1 is a simple and low cost 2-D EIT system. Its accuracy and operation are tested in different conditions. The system is designed to be upgraded to function as a multi-current generator adaptive system. Also by modification of the sampling circuit, SUT-1 will be able to detect the imaginary part of the signal can be detected. For this purpose voltage sampling has to be carried out during zero-crossing instead of peak sampling. The system was tested under in-vitro conditions. In order to perform in-vivo measurement, the IEC-601 safety standard has to be observed. The system needs some changes using an isolation component, e.g. opto-couplers in the data acquisition circuit, which can provide a complete electrical isolation. It is believed that the SUT-1 can be also used for different EIT applications such as industrial process control.

The different hardware parts of an engineered EIT system namely SUT-1 were investigated. SUT-1 also has its own limitations in practical use. Primary studies are under way to increase the SUT-1's capabilities. This can be achieved through the use of better electrodes (e.g. active electrodes), faster data acquisition technique, multi-frequency and real time 3-D image processing. Industrial applications of system for optimizing the metallurgical and chemical processes are some of the other potential applications of SUT-1. The idea of this paper to make available a system design for a simple EIT system and has no claim that this is a state of the art EIT system. For a good reference to EIT hardware we refer to a new book edited by David Holder [19]. The book contains a good overview about the design of EIT instrumentation.
